# Validation of a particle tracking analysis method for the size determination of nano- and microparticles

**DOI:** 10.1007/s11051-017-3966-8

**Published:** 2017-08-04

**Authors:** Vikram Kestens, Vassili Bozatzidis, Pieter-Jan De Temmerman, Yannic Ramaye, Gert Roebben

**Affiliations:** 1 Joint Research Centre (JRC), Directorate Health, Consumers and Reference Materials, European Commission, Retieseweg 111, 2440 Geel, Belgium; 2 Service Trace Elements and Nanomaterials, Veterinary and Agrochemical Research Centre (CODA-CERVA), Groeselenberg 99, 1180 Brussels, Belgium

**Keywords:** Measurement uncertainty, Method validation, Nanoparticles, Particle size analysis, Particle tracking analysis, Reference material

## Abstract

Particle tracking analysis (PTA) is an emerging technique suitable for size analysis of particles with external dimensions in the nano- and sub-micrometre scale range. Only limited attempts have so far been made to investigate and quantify the performance of the PTA method for particle size analysis. This article presents the results of a validation study during which selected colloidal silica and polystyrene latex reference materials with particle sizes in the range of 20 nm to 200 nm were analysed with NS500 and LM10-HSBF NanoSight instruments and video analysis software NTA 2.3 and NTA 3.0. Key performance characteristics such as working range, linearity, limit of detection, limit of quantification, sensitivity, robustness, precision and trueness were examined according to recommendations proposed by EURACHEM. A model for measurement uncertainty estimation following the principles described in ISO/IEC Guide 98-3 was used for quantifying random and systematic variations. For nominal 50 nm and 100 nm polystyrene and a nominal 80 nm silica reference materials, the relative expanded measurement uncertainties for the three measurands of interest, being the mode, median and arithmetic mean of the number-weighted particle size distribution, varied from about 10% to 12%. For the nominal 50 nm polystyrene material, the relative expanded uncertainty of the arithmetic mean of the particle size distributions increased up to 18% which was due to the presence of agglomerates. Data analysis was performed with software NTA 2.3 and NTA 3.0. The latter showed to be superior in terms of sensitivity and resolution.

**Electronic supplementary material:**

The online version of this article (doi:10.1007/s11051-017-3966-8) contains supplementary material, which is available to authorized users.

## Introduction

Achieving accurate particle size results is critical for many different industrial and research applications and processes. For nanoparticles, this demand has become even more important with the adoption of the Recommendation ‘on the definition of a nanomaterial’ (EC 2011) which is based on particle number-based distributions of particle size values. A wide variety of techniques such as scanning and transmission electron microscopy (SEM and TEM), dynamic light scattering (DLS), centrifugal liquid sedimentation (CLS), scanning probe microscopy (SPM) and small-angle X-ray scattering (SAXS) are available for particle characterisation. Particularly in the context of the EC nanomaterial definition, SEM and TEM have attracted most attention as the obtained results are intrinsically number-weighted. The main disadvantages of these electron microscopy-based techniques are the low sample throughput, the poor statistical sampling that results from the relatively low number of particles counted by the analysis, high measurement time and cost, and the high level of operator expertise needed.

Particle tracking analysis (PTA), often referred to as nanoparticle tracking analysis (NTA), is a technique (Malloy and Carr 2006) that intends to overcome several of the limitations mentioned above, i.e. for a single measurement, a significantly higher number of particles can be analysed in typically less than 10 min. As a result, the technique is sometimes promoted as a cost-effective alternative to SEM and TEM analyses. In contrast to the ensemble-based DLS technique, PTA measurements are performed on a particle-by-particle basis and are therefore assumed to be less prone to interference caused by agglomerates or larger particles when present in a heterogeneous test sample.

PTA combines laser light scattering with an optical microscope. The microscope unit is mounted almost perpendicular to the direction of the incident laser beam and is equipped with a camera which records the light scattered by the individual particles that are undergoing Brownian motion. The instrument software tracks the trajectories of the particles detected in the microscope’s field of view and determines their mean-square displacements in two dimensions (Qian et al. 1991). The translational diffusion coefficient of each tracked particle is then computed and converted into a sphere-equivalent hydrodynamic diameter according to the following modified Stokes-Einstein Eq. (1):

1 $$ \overline{\frac{{\left(x,y\right)}^2}{4t}}={D}_{x,y}=\frac{k_{\mathrm{B}}T}{3\pi \eta d} $$

where $\overset{-}{{\left(x,y\right)}^2}$ is the mean-square displacement in two dimensions, *D*_*x*,*y*_ is the translational diffusion coefficient in two dimensions, *k*_B_ is Boltzmann’s constant, *T* is the sample temperature, *t* is the time, *η* is the viscosity of the dispersion medium and *d* is the sphere-equivalent hydrodynamic diameter.

Particular attention must be paid to the fact that the validity of the measurement model, as presented in Eq. (1), is based on a number of assumptions: (1) all particles must be freely and uncorrelated moving in all three dimensions under the influence of Brownian diffusion and (2) particles must be colloidally stable during the measurement time, *t*. In addition, particles with a non-spherical or non-equiaxial shape are subjected to a combination of translational and rotational diffusion. In particular for high-aspect ratio particles (e.g. carbon nanotubes, rod- and plate-like particles), rotational movement causes flickering of the scattered light and this can perturb the tracking and detection of the particles and, as a result, significantly affect the statistical reliability of the measured equivalent hydrodynamic diameter results (Gallego-Urrea et al. 2014). The data analysis algorithms implemented in the particle tracking software which have been used in the presented work do not account for rotational diffusion.

The PTA measurement procedure generally consists of sample preparation, video recording and video analysis. Sample preparation often requires diluting the supplied sample in an aqueous medium such as purified water until the particle concentration is within the recommended range of 10^7^ to 10^9^ particles/mL. According to Krueger et al. (2016), the process that covers the recording and analysis of videos can be prone to bias as various acquisition and analysis parameters must be optimised and defined by the individual analyst. The adjustment of these parameters can significantly impact the shape of the measured size distribution (Filipe et al. 2010). The key parameters for data collection are the gain and the shutter speed of the camera, the capture duration, and the alignment of the sample holder and the optical system, while the main critical parameters for data analysis are the screen gain, detection threshold, the minimum expected particle size and blur (Patois et al. 2012). De Temmerman et al. (2014a) systematically compared PTA results with results from TEM and estimated measurement uncertainties for both sets of data. The reported PTA relative expanded measurement uncertainties were 7%, 9% and 14% for nominal 200 nm and 100 nm polystyrene and 30 nm gold, respectively. These uncertainties only cover measurement variation due to method repeatability and intermediate precision. Sauvain et al. (2017) recently validated a PTA method for measuring the size and number concentration of particles in a biological medium (i.e. exhaled breath condensate). In their validation study, measurements were also performed on nominal 100 nm PSL particles dispersed in pure water and in biological medium. The relative standard uncertainty for precision was estimated as 4.5% (in water) and 8% (in biological medium). However, both De Temmerman et al. and Sauvain et al. did not use certified reference materials for quantifying the aspect of method trueness. No other attempts have been made so far to fully validate the PTA method for particle size analysis using a variety of non-certified and certified reference materials and according to recommendations proposed by EURACHEM (2014).

This study aims to demonstrate, through a validation study, that the PTA method is fit for the purpose of determining selected characteristic values of the number-weighted particle size distributions (PSD) of populations of monomodal and bimodal mixtures of silica and polystyrene particles in aqueous suspension. Silica and polystyrene suspensions were selected as model samples as the particles have a (near-)spherical shape, which is an assumption for the validity of the Stokes-Einstein equation, and because they are available as reference materials in a variety of sizes. Reference materials come with a quantified level of heterogeneity, and this reduces the risk that random variation is interpreted as a method characteristic while in practice it is due to sample heterogeneity. For the three targeted measurands (i.e. mode, median and arithmetic mean of a number-weighted PSD), relative expanded uncertainties up to a maximum of about 10% would be acceptable. Measurement uncertainties in the range of 3% to 16% have been frequently reported for the size results of nanoparticles measured by other techniques such as transmission electron microscopy (De Temmerman et al. 2014a), dynamic light scattering and centrifugal liquid sedimentation (Braun et al. 2011). This study also provides a systematic comparison between results computed by the previous, but still commonly used, software version NTA 2.3 and one of the newer software versions NTA 3.0, which employs an improved data fitting algorithm.

## Materials and methods

### PTA instrument

Optimisation and validation experiments were carried out with NanoSight instruments models NS500 and LM10-HSBF (Malvern Instruments Ltd., Worcestershire, UK). Both PTA instruments are equipped with an optical microscope objective lens of 20x magnification, a laser diode with a wavelength of 405 nm and maximum power output of 65 mW and an Orca-Flash 2.8 scientific complementary metal-oxide-semiconductor (sCMOS) camera (Hamamatsu Photonics, Hamamatsu City, Japan). The sCMOS cameras were operated at 25 frames per second. The field of view of the optical microscopes was calibrated by the manufacturer against the 100 nm size value (as indicated by TEM) of a polystyrene particle standard (Thermo Scientific, Fremont, USA).

The LM10-HSBF device was used for measuring ERM-FD101b during the trueness assessment stage. All other measurements were performed with the NS500 device. Particle size results were calculated from the determined translational diffusion coefficients using a modified version of the Stokes-Einstein formula, Eq. (1) (Filipe et al. 2010). The viscosity of the suspension medium, which was assumed to be that of pure water, was for each analysis automatically adjusted according to the measured temperature. Video files were acquired using the instrument software version NTA 2.3 (Build 33) and were analysed with both software versions NTA 2.3 (Build 33) and NTA 3.0 (Build 69). Apart from the redesigned interface and integrated hardware detection and communication, NTA 3.0 employs an improved and high-resolution finite track length adjustment (FTLA) particle size distribution algorithm which provides a better separation of different particle size populations (Walker 2012). Compared to the algorithm used in NTA 2.3 and earlier versions, the FTLA algorithm (algorithm described by Walker (2012) with some proprietary modifications) is model independent in that there is no assumption that the sample is monomodal. The latest software versions currently available are NTA 3.1 and NTA 3.2. The performance of these software versions was not investigated as they were released by the manufacturer after our validation study. NTA 3.1 and NTA 3.2 use the same FTLA data analysis algorithms as the one implemented in NTA 3.0. Hence, it can be assumed that the measurement uncertainties estimated from the validation data will also be valid for particle size results analysed with NTA 3.1 and NTA 3.2.

### Test materials

Three colloidal silica and ten polystyrene latex (PSL) reference materials were analysed during the optimisation and validation studies. An overview of the different materials and relevant assigned particle size values extracted from the material certificates is given in Table 1.

**Table 1:** Reference materials used during the method optimisation and validation studies, relevant assigned particle size values and associated expanded (*k* = 2) uncertainties

Reference material ID	Supplier’s reference	Particle mass fraction [g/kg]	Method used for value assignment	Assigned particle size value (± uncertainty) or range [nm]
ERM-FD304	ERM-FD304	2.5	DLS (cumulants algorithm)^a^	42.1 ± 0.6
			SEM and TEM^c^	27.8 ± 1.5
ERM-FD101b	ERM-FD101b	2.5	DLS (cumulants algorithm)^a^	89.5 ± 2.3
			DLS (distribution calculation algorithms)^b^	93 ± 4
			SEM and TEM^c^	83.7 ± 2.2
			SEM and TEM^d^	83.5 ± 2.2
			PTA^e^	82 ± 4*
			PTA^f^	87 ± 4*
			PTA^g^	82 ± 4*
ERM-FD102	ERM-FD102	Size class A—10.0 (nominal 20 nm) Size class B—2.5 (nominal 80 nm)	DLS (distribution calculation algorithms)^b^	17.8 ± 1.5 88.5 ± 2.2
			SEM and TEM^c^	18.2 ± 1.6 84.0 ± 2.1
			SEM and TEM^d^	18.3 ± 1.7 83.3 ± 2.3
			PTA^e^	78 ± 5
			PTA^f^	82 ± 4
			PTA^g^	79.2 ± 2.2
PSL-21	3020A	10	DLS	21 ± 1.5
PSL-31	3030A	10	DLS	31 ± 3
PSL-41	3040A	10	DLS	41 ± 4
PSL-46	3050A	10	TEM	46 ± 2
			DLS	45–51
PS-59	3060A	10	TEM	59.0 ± 2.5
		10	DLS	58–65
PSL-81	3080A	10	TEM	81 ± 3
			DLS	79–84
PSL-100	3100A	10	TEM	100 ± 3
			DLS	98–103
PSL-125	3125A	10	TEM	125 ± 3
			DLS	120–130
PSL-147	3150A	10	TEM	147 ± 3
			DLS	146–153
PSL-200	3200A	10	TEM	200 ± 6
			DLS	196–206

^*^Certified values used for trueness assessment

^a^Sphere-equivalent intensity-weighted harmonic mean hydrodynamic diameter

^b^Sphere-equivalent intensity-weighted arithmetic mean hydrodynamic diameter

^c^Area-equivalent number-weighted circular modal diameter

^d^Area-equivalent number-weighted circular median diameter

^e^Sphere-equivalent number-weighted modal hydrodynamic diameter

^f^Sphere-equivalent number-weighted arithmetic mean hydrodynamic diameter

^g^Sphere-equivalent number-weighted median hydrodynamic diameter

The colloidal silica reference materials were provided by the Directorate-General Joint Research Centre (JRC) of the European Commission (Geel, Belgium). While ERM-FD304 and ERM-FD101b have a monomodal PSD, the PSD of ERM-FD102 has two main modes which are further referred to as size class A and size class B. ERM-FD102 also contains a third minor fraction of particles with diameters in the range of 40 nm to 50 nm. However, the distribution characteristics of this population of particles were not assigned. All three colloidal silica materials are certified reference materials, but only ERM-FD101b comes with certified values for the mode, median and arithmetic mean of the particle number-weighted size distributions obtained by PTA. Hence, only PTA-certified values assigned to ERM-FD101b were used for assessing the trueness of the validated PTA method. ERM-FD304 and ERM-FD102 are certified reference materials too because some of the assigned values have been obtained with validated methods (e.g. DLS, SEM, TEM). However, for ERM-FD304, no particle size value has been assigned for PTA. For ERM-FD102, the assigned indicative values for PTA originate from only three laboratories. A minimum of six laboratories is usually required for the assignment of a certified value. Therefore, in this validation study, ERM-FD304 and ERM-FD102 were regarded and used as (non-certified) reference materials.

The monomodal PSL materials with assigned diameters ranging from 21 nm to 200 nm were purchased from Thermo Scientific (Fremont, USA). This size range was particularly chosen as it fairly matches the size range of manufactured nanoparticles used in food, feed and cosmetic products (Contado 2015; Peters et al. 2016). To evaluate the relative resolution, different bimodal mixtures of different peak ratios were prepared from selected monodisperse PSL materials (Table 2). The nominal particle number concentration was calculated from the theoretical mass fraction (10 g/kg), the particle density (1.05 g/cm^3^) and the assigned particle diameter.

**Table 2:** Specifications of the bimodal PSL mixtures

Test material ID	Monodisperse PSL materials		Nominal particle number concentration [×10^7^ particles/mL]	
	Fraction 1	Fraction 2	Fraction 1	Fraction 2
PSL-50-100_2:1	PSL-50	PSL-100	7.6	3.8
PSL-50-100_4:1	PSL-50	PSL-100	15.3	3.8
PSL-60-100_1.2:1	PSL-60	PSL-100	4.4	3.8
PSL-60-100_2.3:1	PSL-60	PSL-100	8.8	3.8
PSL-50-80_3.6:1	PSL-50	PSL-80	135.0	37.3
PSL-80-100_3.1:1	PSL-80	PSL-100	37.3	11.9
PSL-50-60_1.7:1	PSL-50	PSL-60	30.6	17.7
PSL-50-60_5.2:1	PSL-50	PSL-60	91.8	17.7

### Sample preparation

The as-received reference materials were diluted in purified water. The purified water was prepared from regular tap water that had subsequently undergone reverse osmosis, UV-irradiation and filtration through a membrane with nominal pore sizes of 220 nm (Merck Millipore Inc., Billerica, MA, USA). The purified water, which had a resistivity of 18.2 MΩ cm at 25 °C, was additionally passed through a membrane filter with nominal pore sizes of 100 nm to remove particles that were not retained by the 220 nm pore size filter of the purification system. Before each series of measurements, the cleanliness of the purified water was checked at the required instrument settings to ensure that the concentration of particles present in the purified water was below the limit of detection.

## Results and discussion

The method validation study and measurement uncertainty estimations were carried out according to the EURACHEM (2014) and ISO/IEC Guide 98-3 (2008) guidelines. The method performance characteristics which were considered to be relevant for the PTA method were selectivity, calibration/working range, limit of detection (LOD), limit of quantification (LOQ), sensitivity, robustness and accuracy (i.e. precision and trueness). The measurands of interest were the mode, median and arithmetic mean of the particle number-weighted distribution of sphere-equivalent hydrodynamic particle diameter values.

### Selectivity

Selectivity relates to the extent to which a method can be used to determine particular analytes in mixtures or matrices without significant interferences from other components of similar behaviour.

In this study, all test samples were diluted and dispersed in purified water. Due to their difference in refractive index and their very small size, water molecules scatter light much less than silica and polystyrene particles, and it can, therefore, be assumed that the water as background does not significantly interfere with the light scattered by the dispersed particles.

For heterogeneous samples, the PTA method is not selective because the instrument detects all scattered light regardless of the kind of particle that is the scattering source. Agglomerates and other undesired large particles can scatter light more intensively and can be the cause of interferences, i.e. the clouding of underlying small particles, and can drastically perturb the PSD.

### Calibration, working range, LOD and LOQ

The PTA working range (and LOD and LOQ) depends both on particle size and particle concentration. According to the instrument manufacturer, the PTA method can measure the size of particles with diameters of 10 nm to 2 μm and particle concentrations in the range of 10^7^ to 10^9^ particles/mL. The influence of the particle concentration on the determination of the mean, median and modal values of the PSDs of PSL-100 is discussed and shown in the Electronic supplementary information (ESI). These results show that within a particle concentration range of 1.9 × 10^6^ to 1.9 × 10^9^ particles/mL, both the NTA 2.3 and NTA 3.0 software calculate mean and modal particle size results that agree with the assigned reference value within ±1.5% (relative standard uncertainty).

The actual lower limit of the working range for particle size depends on the light scattering properties (e.g. refractive index contrast) of the particles and the type of camera used. On the other hand, the upper limit of the range is defined by the effective density of the particles which determines whether during the measurement the particles remain in suspension due to Brownian motion or whether sedimentation forces are dominant. For the presented PTA method, the working range for particle size measurements was confirmed and fixed based on the modal values of the particle number-weighted size distributions obtained for the reference materials listed in Table 1.

Particle sizing with PTA is an absolute method; in other words, it relies only on the first principles captured in Eq. (1). As a result, there is no need for the analyst to calibrate the instrument’s signal response with particle size calibrants. However, the distances travelled by the particles and measured by the instrument software need to be correctly measured, and for this, a calibration constant has been determined by the manufacturer. PSL particles of 100 nm in diameter or an optical diffraction grid is typically used by the manufacturer to calibrate the length scale of the optical microscope’s field of view and to convert pixels into the SI unit of length (metre). The instrument manufacturer states that in the case of the calibration grid, a distance of 100 μm representing about 600 pixels is measured, and the user-to-user variation is about 2 pixels or 0.4% at the extreme. No further details are available from the manufacturer concerning the calibration with the 100 nm PSL particles.

**Fig. 1:**
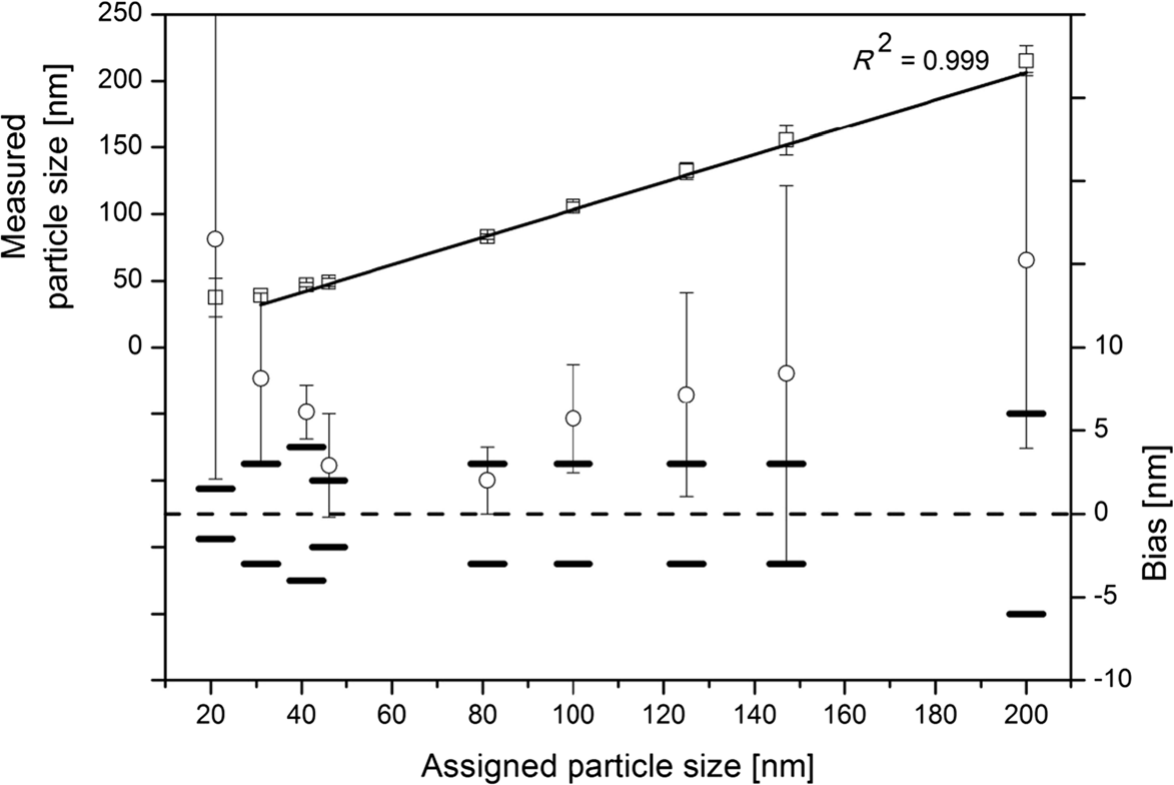
Results of linear relation between assigned and measured particle size (*squares*; *left y*-axis); bias (*circles with error bars* indicating standard deviations; *right y*-axis) between the mean measured modal particle diameter and particle diameter assigned by TEM or DLS (in the case of PSL-21, PSL-31 and PSL-41). Expanded uncertainties associated with the assigned values are indicated with *en dash* symbols (**–**)

As can be seen from Fig. 1, measurements on eight different PSL particle size standards indicate that the one-point calibration yields a linear relationship (*R* ^2^ = 0.999) between the means of the modal diameter results measured with software NTA 2.3 and the particle diameter values assigned to the tested PSL materials. The PSDs obtained for the smallest PSL (PSL-21) were multimodal, and the peak that corresponded to the individual non-agglomerated particles could not always be unambiguously determined due to the method’s limit of quantification. Therefore, the linear fit does not include the results of PSL-21. The results obtained on the other PSL materials confirm the working range for monodisperse PSL particles with diameters between 31 nm and 200 nm. The relative standard deviations (RSD) that were calculated for each set of nine replicate results varied between 2.4% (PSL-81) and 7.3% (PSL-147). A significantly higher RSD of 13.1% was calculated for the results obtained for PSL-31. Since all measurement results were achieved under intermediate precision conditions, the RSD values give a first estimate of the precision of the PTA method. Considering the target relative expanded (*k* = 2, confidence level of about 95%) uncertainty of 10% (which is equivalent to a relative standard uncertainty of 5% at a confidence level of 68%), the maximum relative standard uncertainty for precision should ideally be in the range of about 3% to 4%. For PSL-31, the RSD value of 13% is significantly higher than 4%, while the RSD values of the other materials vary from about 2% to 7%. It must be stressed that these measurements were not carried out according to a nested study design and that possible sample-to-sample heterogeneity may also be included in the variances. Also, no trends between the RSD and particle size values were observed. Hence, we regard the lower limit (31 nm) of the range of the linear fit as the lower LOD and 41 nm as the lower LOQ. For PSL-200, an RSD value of 5.3% was calculated, fixing both the upper LOD and upper LOQ at 200 nm.

The second set of data presented in Fig. 1 shows the absolute difference (i.e. bias) between the mean measured modal values and the theoretical values and their associated expanded uncertainties. For the tested PSL materials, the PTA size values were systematically higher than the size values that were assigned by the manufacturer using TEM and DLS. This bias may be caused by a combination of different factors. On the one hand, differences between particle size results can stem also from the different physical principles and data analysis algorithms of the measurement techniques (Kestens et al. 2016). On the other hand, as highlighted by others, polymeric particles can shrink from prolonged exposure to the focussed electron beams of electron microscopes (McDonald et al. 1977).

**Fig. 2:**
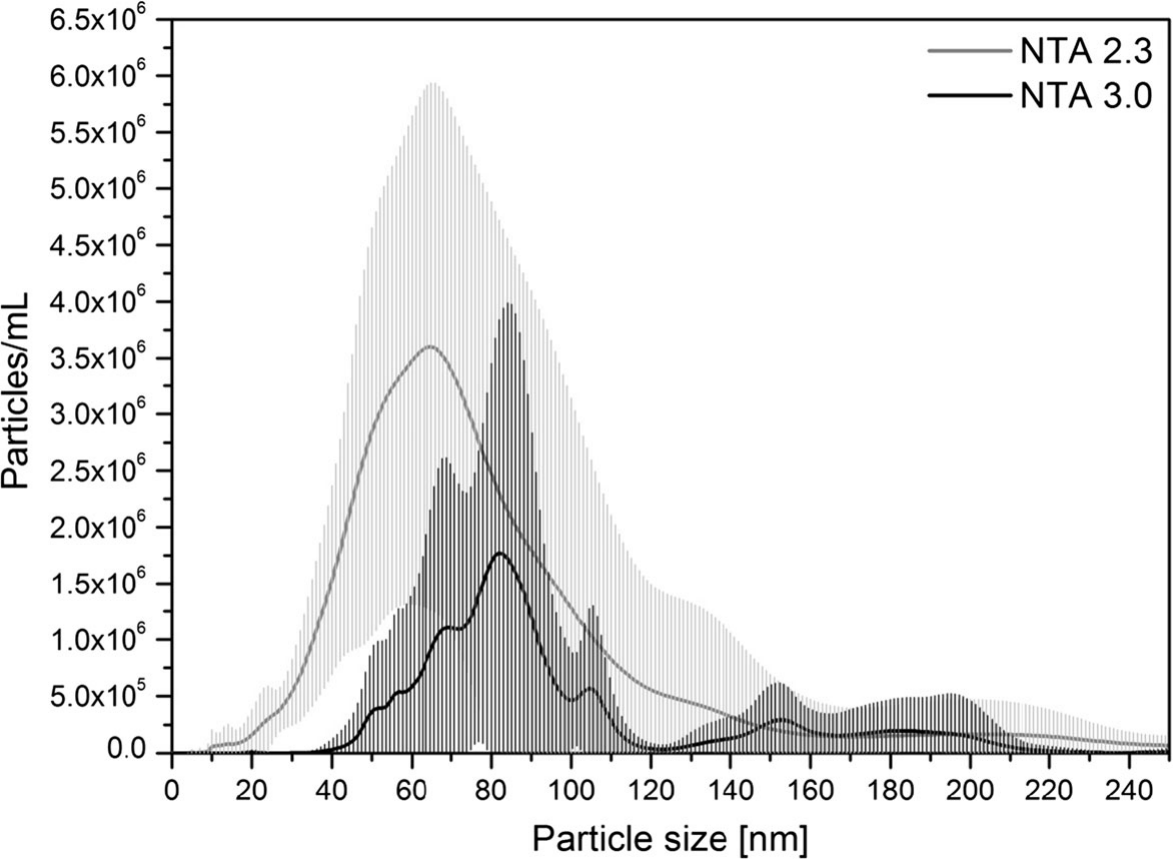
Mean number-weighted PSD representing of ERM-FD304 obtained by PTA. *Shaded areas* correspond to the standard deviation of 56 analyses of the same material

**Fig. 3:**
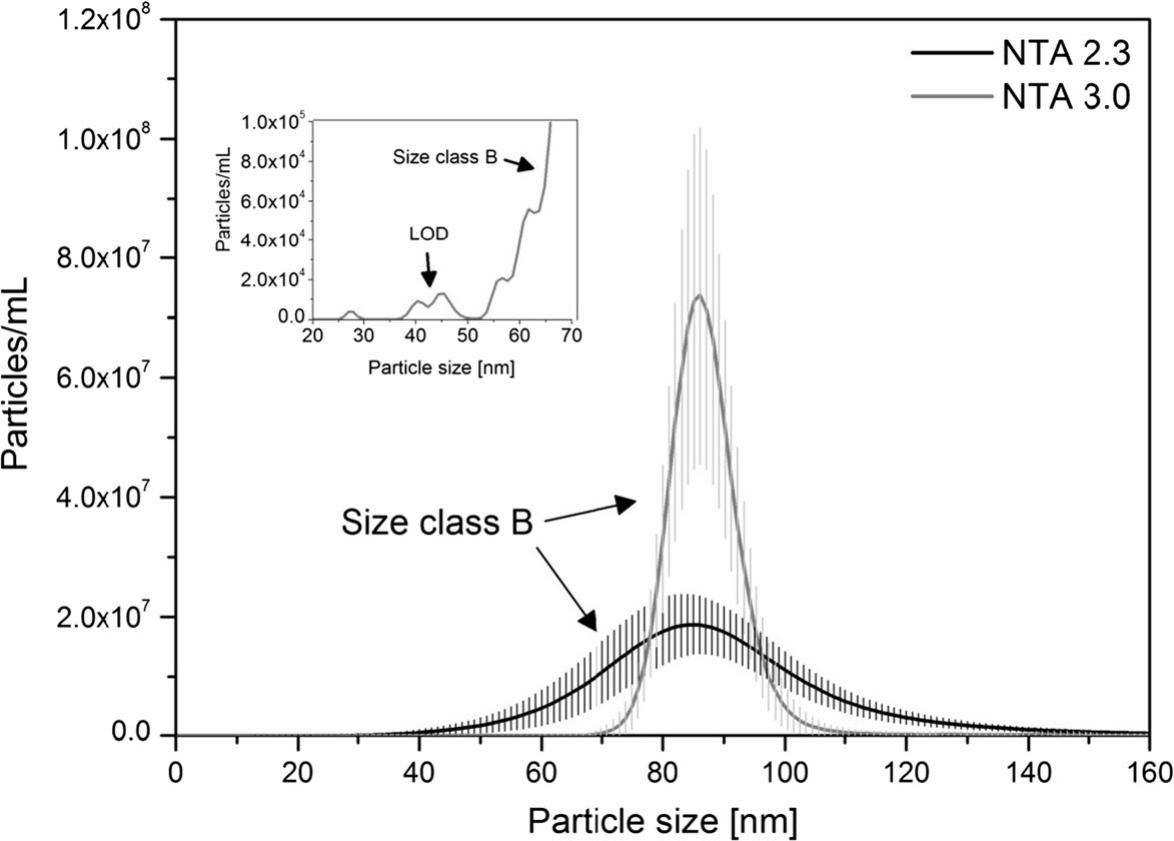
Mean number-weighted PSD of ERM-FD102 obtained by PTA. *Shaded areas* correspond to the standard deviation of 54 analyses of the same material. The *inset figure* shows a zoomed region of the sub-70 nm particle size range indicating the presence of the third minor particle population (*error bars* are omitted for clarity)

At this stage, only two colloidal silica materials were tested: ERM-FD304 and ERM-FD102. As can be seen from the large error bars (or shaded areas) in Fig. 2, ERM-FD304 could not be analysed with an acceptable degree of precision. Also, the modal values of the PSDs were significantly larger than the certified (42.1 nm for DLS) and indicative (27.8 nm for electron microscopy) values. Similar observations were recently made by Tuoriniemi et al. (2014) and Nicolet et al. (2016) who reported biased PTA results of about 67 nm and 87 nm for silica nanoparticles of nominally 35 nm and 48 nm in diameter, respectively. Nicolet et al. suggested that the systematic error was due to the method’s insensitivity to the small particles in the sample and the counting of particle clusters instead. Another possible explanation for the bias is that the laser beam exhibits a Gaussian intensity profile when illuminating the sample. Large and strongly scattering particles at the less intense beam edge may remain detectable and quantifiable, while the scattered light of small particles with a low refractive index contrast may drop below the LOQ (Malloy and Carr 2006). An acceptable degree of precision was obtained for the results that were associated with the peak of the size class B fraction of ERM-FD102 (Fig. 3). The size class A fraction could hardly be detected (see insert in Fig. 3). Only for few PSDs, a very small peak between 25 nm and 30 nm, which may present the nominal 20 nm particles, could be observed. The third minor particle population could be seen for practically all PSDs in the size range of about 35 nm to 55 nm. The mode of that fraction could, however, not be determined accurately. For silica particles, the lower LOD and LOQ can be concluded as about 40 nm (from ERM-FD304) and about 80 nm (from ERM-FD102), respectively. In agreement with the approaches used by Tsai et al. (2011) and De Temmerman et al. (2014a), this lower LOD agrees well with a theoretical LOD of about 33 nm which is calculated by comparing the scattered light intensity ratios for polystyrene particles of 31 nm in diameter and a refractive index of 1.59, and silica particles with a refractive index of 1.54.

### Sensitivity and resolution

The sensitivity of the PTA method to detect and quantify, within its working range, different particle populations was investigated by preparing different bimodal mixtures of monodisperse PSL materials. Here, the sensitivity is quantitatively expressed in terms of resolution (*R*_s_), which is a useful measure of the effectiveness of separation of two neighbouring peaks. An overview of the different valid approaches for calculating *R*_s_ values is provided in the ESI. The *R*_s_ values listed in Table 3 were calculated according to the resolution function which is given by Eq. S2. The necessary peak-related characteristic values such as the peak widths measured at half-height were estimated by fitting the experimental data of each peak, or the resolved parts thereof, with simulated monomodal data that followed a Gaussian function (Fig. S5).

**Table 3:** Local maxima (mean ± SD in nm) of the bimodal peaks of the number-weighted PSDs of PSL bimodal mixtures and associated *R*_s_ values

Test material ID	Number of replicates	NTA 2.3			NTA 3.0		
		Fraction 1	Fraction 2	*R*_s_	Fraction 1	Fraction 2	*R*_s_
PSL-50-100_2:1	3	55 ± 3	95 ± 3	0.7	57 ± 5	105 ± 3	2.0
PSL-50-100_4:1	3	51 ± 3	100 ± 2	0.9	53 ± 2	100 ± 4	1.9
PSL-60-100_1.2:1	4	62 ± 5	98 ± 3	0.7	65 ± 3	102 ± 4	1.4
PSL-60-100_2.3:1	4	63 ± 2	94 ± 5	0.5	65 ± 4	97 ± 5	1.2
PSL-50-80_3.6:1	9	<LOD	78 ± 2	N.A.	53 ± 13	83 ± 4	1.0
PSL-80-100_3.1:1	3	86 ± 3	<LOD	N.A.	84 ± 1	100 ± 2	0.5
PSL-50-60_1.7:1	3	<LOD	59 ± 2	N.A.	<LOD	63 ± 1	N.A.
PSL-50-60_5.2:1	3	48 ± 1	<LOD	N.A.	52^a^	61^a^	0.5^a^

*N.A.* not applicable as only one fraction could be detected

^a^Based on a single measurement result

Different method parameters can have an influence on the resolution. This study only compares the effect of the two different PSD algorithms used in software versions NTA 2.3 and NTA 3.0.

**Fig. 4:**
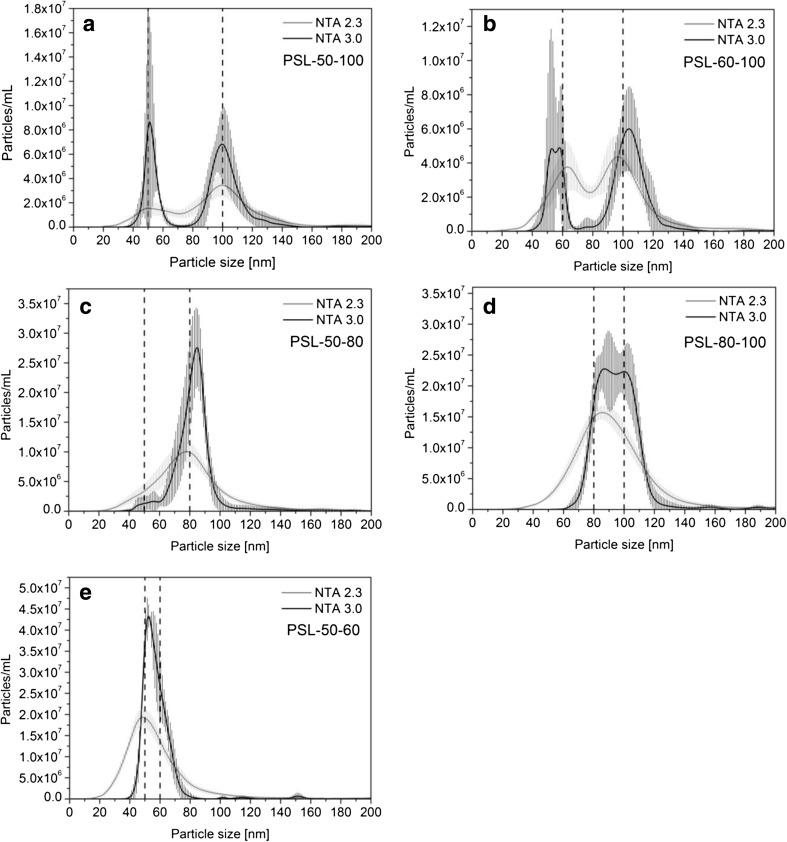
Mean number-weighted PSDs of bimodal PSL mixtures obtained by PTA. *Shaded areas* correspond to the standard deviation of four analyses of the same sample, *vertical dashed lines* indicate the nominal particle size value of each population

A complete peak separation could be obtained for PSL-50-100 and PSL-60-100 when using NTA 3.0 while NTA 2.3 only achieved marginal peak separations (Fig. 4a, b). For PSL-50-80, PSL-80-100 and PSL-50-60, NTA 2.3 could only determine monomodal PSDs of which the modes were shifted towards one of the two particle populations (Fig. 4c–e). NTA 3.0 could marginally separate the peaks of the PSL-50-80 and PSL-80-100 particles, allowing reliable determination of the associated local maxima. For the PSL-50-60 material, only one of the three acquired PSDs showed the presence of the two particle populations.

In general, it can be concluded that thanks to the FTLA algorithm, software NTA 3.0 provides superior resolution compared to software NTA 2.3. *R*_s_ values of ≥1.2 correspond to complete peak separation, and a complete peak analysis can be performed using simple statistics. *R*_s_ values of <1.2 indicate marginal peak separation, and the partially resolved particle populations can only be approximatively characterised using the local maxima.

### Precision, trueness and measurement uncertainty estimation

The accuracy of the PTA data (i.e. the combination of precision and trueness) was determined by measuring PSL-46, PSL-100, ERM-FD102 and ERM-FD101b. The first three reference materials were used for assessing the precision of the method by performing measurements under repeatability and intermediate precision conditions according to a nested design; a total of 20 aliquots were analysed over the course of 5 days (four aliquots per day) by one operator using the NS500 device. The trueness of the method was investigated by analysing nine replicates of ERM-FD101b over the course of 3 days (three aliquots per day) by another operator using the LM10-HSBF instrument. Each single aliquot result is the mean of three results obtained on three sub-samples that were consecutively loaded into the measurement cell and analysed under repeatability conditions. The precision and trueness of the PTA method was quantified for the modal, median and arithmetic mean central values of the number-weighted PSDs. An overview of the obtained results is given in Table 4. In addition to the modal, mean and median (*d*_50_) values, the instrument software also computes cumulative particle size results at the 10th (*d*_10_) and 90th (*d*_90_) percentiles. The percentile results can be used for calculating the span (i.e. (*d*_90_ − *d*_10_)/*d*_50_), which is a dimensionless measure of the distribution width relative to the median. The breadth of the PSD curve around the mean is given by 1× standard deviation (SD). These additional results are only informative, i.e. the performance of the PTA method to quantify a distribution of particle size results was outside the scope of the given validation study.

**Table 4:** Characteristic values (mean ± SD of aliquot means, in nm) of monomodal number-weighted PSDs

Peak characteristic	NTA 2.3				NTA 3.0			
	PSL-46	PSL-100	ERM-FD101b	ERM-FD102 (size class B)	PSL-46	PSL-100	ERM-FD101b	ERM-FD102 (size class B)
Mean	63 ± 7	119 ± 12	88 ± 1	94 ± 4	60 ± 7	111 ± 7	84 ± 1	91 ± 3
Mode	49 ± 3	106 ± 4	82 ± 1	85 ± 3	51 ± 1	106 ± 4	87 ± 1	86 ± 1
Median (*d*_50_)	54 ± 4	111 ± 4	84 ± 1	88 ± 2	51 ± 2	106 ± 4	78 ± 1	86 ± 1
*d*_10_	37 ± 4	88 ± 4	N.A.	67 ± 2	44 ± 2	98 ± 4	N.A.	79 ± 1
*d*_90_	97 ± 17	158 ± 39	N.A.	120 ± 5	79 ± 25	132 ± 39	N.A.	95 ± 3
Span	1.1 ± 0.2	0.6 ± 0.1	N.A.	0.6 ± < 0.1	0.7 ± 0.4	0.3 ± 0.2	N.A.	0.2 ± <0.1
Breadth	34 ± 12	35 ± 14	N.A.	35 ± 9	29 ± 18	21 ± 16	N.A.	27 ± 13

*N.A.* no data available

In-house method validation does typically not address method reproducibility or between-laboratory precision. In 2013, four rounds of interlaboratory comparison (ILC) studies were organised by Hole et al. (2013). A total of 12 laboratories participated in the different ILCs. While no measurement instructions were provided to the ILC participants during the first ILC, detailed protocols for sample preparation, video acquisition and data analysis were disseminated to the participants of the next ILC rounds. For a nominal 100 nm polystyrene reference material, and by using robust statistics, the reproducibility, expressed as a relative standard deviation, could be improved from 40% (ILC 1) to about 10% (ILC 2) and 3% (ILC 3 and ILC 4). The aspect of reproducibility is particularly relevant if data obtained by different laboratories are to be compared. Since the ILC results (Hole et al. 2013) were obtained on a reference material similar to those used during our validation study, it can be assumed that the relative standard deviation of 3% is a reliable estimate of the relative standard uncertainty for method reproducibility. In addition to reproducibility, precision also includes repeatability and intermediate precision. Repeatability indicates the closeness of results performed over a short period, using the same instrument and method, and performed by the same operator. On the other hand, intermediate precision mainly reflects the closeness of results obtained over different sample runs and days.

The relative standard uncertainty for precision (*u*_prec_) was estimated following Eq. (2). It must be emphasised that for our validated PTA method, the standard uncertainty for precision will only be valid for the mean calculated from the results of three replicates (aliquots) obtained on a single day by one laboratory, which is a typical measurement scheme applied by laboratories, hence the choice of the values for *n*_rep_, *n*_day_ and *n*_lab_ in the denominators in Eq. (2).

2 $$ {u}_{\mathrm{prec}}=\sqrt{\frac{RSD_{\mathrm{r}}^2}{n_{\mathrm{r}\mathrm{ep}}}+\frac{RSD_{ip}^2}{n_{\mathrm{day}}}+\frac{RSD_{\mathrm{R}}^2}{n_{\mathrm{lab}}}}=\sqrt{\frac{RSD_{\mathrm{r}}^2}{3}+\frac{RSD_{ip}^2}{1}+\frac{RSD_{\mathrm{R}}^2}{1}} $$

The RSD value for reproducibility (RSD_R_) is taken from the fourth ILC study that was conducted in frame of the EU FP7 QualityNano project (Hole et al. 2013), and the values for repeatability (RSD_r_) and intermediate precision (RSD_ip_) are calculated based on Eqs. (3) and (4):

3 $$ {RSD}_{\mathrm{r}}=100\cdotp \frac{\sqrt{MSW}\ }{y_{\mathrm{m}}} $$

4 $$ {RSD}_{ip}=100\cdotp \frac{\sqrt{\frac{MSB-MSW}{n}}}{y_{\mathrm{m}}} $$

where MSW and MSB are the mean of squares within and between measurement days, respectively, calculated using one-way analysis of variance (ANOVA); *n* is the number of aliquots per day; and *y*_m_ is the mean value of the replicate particle size results.

If MSW > MSB, then RSD_ip_ cannot be calculated due to the negative argument under the square root. In such situation, the value for RSD_ip_^*^ is calculated alternatively according to Eq. (5) (Federer 1968).

5 $$ {RSD}_{ip}^{\ast }=100\cdotp \frac{\sqrt{\frac{\left(MSB-MSW\right)+MSW}{n}\cdotp {e}^{-\left(\frac{MSB}{MSW}\right)}}}{y_{\mathrm{m}}} $$

For each measurand (mode, median and mean), the trueness of the method was quantitatively assessed in terms of experimental bias (Δ_bias_), which is the absolute difference between the certified PTA values of ERM-FD101b and the mean values of the replicate measurement results. The assessment, including the estimation of uncertainty for method trueness (*u*_t_), was performed according to the procedures described in ERM Application Note 1 (Linsinger 2010). The trueness assessment study can be split in three distinct steps.

During the first step, MSB and MSW were calculated for each measurand from the 3 × 3 data matrix using one-way ANOVA. The relative standard measurement uncertainty (*u*_meas_) associated to the mean of the results of the nine replicates was calculated using Eq. (6). The RSD values for repeatability and intermediate precision were again calculated using Eqs. (3)–(4).

6 $$ {u}_{\mathrm{meas}}=\sqrt{\frac{RSD_{\mathrm{r}}^2}{3}+\frac{RSD_{ip}^2}{3}+\frac{RSD_{\mathrm{R}}^2}{1}} $$

At first sight, Eqs. (2) and (6) are almost identical with the only difference being the value of *n*_day_. In Eq. (6), the value of 3 for *n*_day_ is, however, essential because *u*_meas_ must reflect the three measurement days during which the CRM was measured while *u*_prec_ will be used for estimating the uncertainty of measurement results on routine samples obtained during only 1 day.

In the second step, the relative standard uncertainty for trueness (*u*_t_) is estimated by combining the relative standard uncertainty (*u*_CRM_) of the certified value and the value of *u*_meas_ (Eq. 7).

7 $$ {u}_{\mathrm{t}}=\sqrt{u_{\mathrm{meas}}^2+{u}_{CRM}^2} $$

In the third and final step, the significance of the experimental bias is evaluated in absolute terms against *u*_t_, i.e. if Δ_bias_ ≤ 2 × *u*_t_, then it can be concluded that the bias is not statistically significant.

As shown in Table 5, no significant biases were obtained for ERM-FD101b results analysed with software NTA 2.3 and NTA 3.0. While the biases are not significant, it was observed that the biases for results analysed with NTA 3.0 were significantly higher than those from NTA 2.3. The certified PTA values assigned to ERM-FD101b are based on results from an interlaboratory comparison study (Ramaye et al. 2016) during which laboratories analysed results using both NTA 2.3 and NTA 3.0 software. During that ILC study, results from NTA 2.3 and NTA 3.0 agreed well. As a result, there is no firm indication that the two software versions provide systematically different results.

**Table 5:** Trueness assessment using ERM-FD101b (results in nm)

	NTA 2.3			NTA 3.0		
	Mode	Median	Mean	Mode	Median	Mean
Certified value	82	82	87	82	82	87
Standard uncertainty of certified value (*u*_CRM)_	2	2	2	2	2	2
Mean measured value	82.3	83.8	87.8	87.0	77.7	84.1
Measurement uncertainty (*u*_meas_)	2.7	2.7	2.8	2.6	2.6	2.7
Trueness uncertainty (*u*_t_)	3.4	3.3	3.5	3.3	3.3	3.4
Absolute bias (Δ_bias_)	0.3	1.8	0.8	5.0	4.3	2.9
Significant?	No	No	No	No	No	No

The expanded measurement uncertainties (*U*) were finally obtained by combining the individual standard uncertainty contributions from precision and trueness and by multiplying with a coverage factor of *k* = 2 (for approximately 95% confidence interval) according to Eq. (8). Note that the uncertainty related to the length-scale calibration of the microscope’s field of view is considered negligible compared to the uncertainties from trueness and precision and is, therefore, not included in the uncertainty budget.

8 $$ U=k\cdotp \sqrt{u_{\mathrm{prec}}^2+{u}_{\mathrm{t}}^2} $$

An overview of the standard and expanded measurement uncertainties for the mode, arithmetic mean and median of the particle number-weighted PSDs is given in Tables 6, 7 and 8.

**Table 6:** Relative standard and expanded measurement uncertainties (%) for modal particle size results

Uncertainty component	NTA 2.3			NTA 3.0		
	PSL-46	PSL-100	ERM-FD102 (size class B)	PSL-46	PSL-100	ERM-FD102 (size class B)
Precision (*u*_prec_)	5.3	3.8	3.7	3.3	3.6	3.1
Trueness (*u*_t_)	4.1	4.1	4.1	4.0	4.0	4.0
Expanded (*U*)	13.2	11.2	11.0	10.3	10.8	10.2

**Table 7:** Relative standard and expanded measurement uncertainties (%) for mean particle size results

Uncertainty component	NTA 2.3			NTA 3.0		
	PSL-46	PSL-100	ERM-FD102 (size class B)	PSL-46	PSL-100	ERM-FD102 (size class B)
Precision (*u*_prec_)	8.2	4.2	4.2	8.0	5.0	3.5
Trueness (*u*_t_)	4.0	4.0	4.0	3.9	3.9	3.9
Expanded (*U*)	18.2	11.6	11.6	17.8	12.7	10.6

**Table 8:** Relative standard and expanded measurement uncertainties (%) for median particle size results

Uncertainty component	NTA 2.3			NTA 3.0		
	PSL-46	PSL-100	ERM-FD102 (size class B)	PSL-46	PSL-100	ERM-FD102 (size class B)
Precision (*u*_prec_)	5.4	3.9	3.5	4.0	4.0	3.1
Trueness (*u*_t_)	4.0	4.0	4.0	4.0	4.0	4.0
Expanded (*U*)	13.6	11.2	10.8	11.4	11.3	10.2

When estimating measurement uncertainties from method validation data, the main challenge is often related to the trueness assessment because, in the absence of suitable CRMs, analysts typically use either commercially available non-certified RMs or prepare their own working standards by spiking. The possible risk of applying such approaches is that the true bias cannot be reliably discerned because the measured differences are often a reflection of the contrasting measurement principles of the different methods mostly used for characterisation and/or assigning reference values. In this validation study, we had the advantage of having a fit-for-purpose CRM (ERM-FD101b) available that has been certified using PTA.

As can be seen from the presented uncertainty budgets, the PTA method has a precision in the range of about 3% to 5%. However, as indicated by the *d*_90_ values (Table 4), PSDs were sometimes positively skewed due to the presence of agglomerates. The formation of agglomerates can be triggered by the reduced surfactant concentration which is a direct consequence of the high dilution factors required for PTA. In particular for PSL-46, of which its nominal particle size approaches the lower LOQ of 41 nm and where, because of the particle size its sixth power relationship, the agglomerates scatter light about 35 times more intensely than the smaller primary particles, it is observed that NTA 3.0 offers a much better repeatability (~3%) than NTA 2.3 (~6%). This can be attributed to the superior resolution of the FTLA algorithm. Also, a lack of precision is mostly pronounced when evaluating mean particle size results. This is logical as the mean or central gravity value, which is calculated from the entire peak, can be significantly affected by the agglomerates present in the higher percentiles of the distribution. For the tested PSL materials, the small peaks of the agglomerates were only marginally separated from the main peak of the primary particles, and the PTA software only calculated a global mean value rather than a mean value of the primary particles alone. In order to obtain a better precision for the arithmetic mean particle size measurand, one could consider either evaluating the PSDs manually or applying an automatic particle size cut-off value in the video analysis software. Such interventions should, however, be executed with great care as it may also eliminate larger primary particles from the PSD.

Overall, it is safe to state that the validated PTA method can be used to measure the modal and median particle size values of monodisperse suspensions of polystyrene and silica with an accuracy of about 11% which is only slightly above the pre-defined target of 10%. When approaching the lower LOQ, the measurement uncertainty increases to about 14%. In addition, when using the software NTA 3.0, mixtures of different particle populations can be readily resolved and quantified provided the particle size and concentration of the two individual populations is within the validated working ranges and the ratio of small to big particles is at least 1.25—that is, sample mixtures in which the modal size of the smallest particles is about 25% smaller than the modal size of the biggest particles.

### Robustness

As part of the method optimisation process (see ESI), the effect of selected critical method parameters such as the dilution factor, temperature, camera gain and shutter speed was systematically investigated for PSL-100.

Figure S2 shows that the calculated particle size and the measurement temperature are linearly correlated and that the modal size results evaluated with NTA 2.3 and obtained at a temperature of 24 °C, or higher, agreed best with the size range assigned to PSL-100. For results analysed with NTA 3.0, a good agreement was found for the temperature range of 20 °C to 27 °C. Temperature control is usually more difficult for measurements performed near ambient conditions than for measurements at a slightly higher temperature. Therefore, it was decided to perform the actual validation measurements in the range of 24 °C to 26 °C.

A second key parameter that is related to the video recording process is the setting of the camera gain. The camera gain defines the sensitivity of the camera. The calculated particle size value decreased with increasing camera gain (Fig. S3). This trend is most pronounced for results computed with software NTA 2.3. For modal size values analysed with NTA 2.3 and NTA 3.0, a camera gain value up to 250 and 500 yielded acceptable results.

The shutter speed defines the times the camera shutter is open. During the optimisation process, the shutter speed was varied from 12 ms to 1500 ms (Fig. S4). No significant effect between particle size and shutter speed range was observed, neither for data analysed with NTA 2.3 nor for data analysed with NTA 3.0.

Other parameters which may have an effect on the measurement result, and which were not considered during the method optimisation stage, were the video capture time and the detection threshold. Both parameters were already studied by Gross et al. (2016) using a monodisperse PSL material with nominal diameter of 600 nm and bovine serum albumin particles with a size of about 200 nm. The experiments conducted by Gross et al. demonstrated that the modal particle size values that were analysed at different capture times (30 s to 215 s) and different detection thresholds (values 5 to 10) did not significantly differ from each other and from the assigned size value.

To ensure the robustness of the validated PTA method, different experimental parameters (Table 9) were deliberately varied during the analysis of PSL-46, PSL-100 and ERM-FD102. All other parameters were either kept constant (e.g. frame rate, bin width of 1 nm) or were set to automatic (e.g. blur, maximum jump distance) which then allowed the software programme to determine the optimal conditions for the given sample aliquot. It can be concluded that the validated method is robust against the applied parameter variations and that any potential effect is covered by the repeatability and intermediate precision of the validated method.

**Table 9:** Method parameters and parameter values used during the PTA validation study

Parameter	Level
Sample preparation—dilution factors	
PSL-46	1 × 10^5^, 5 × 10^5^ and 1 × 10^6^
PSL-100	1 × 10^5^, 5 × 10^5^ and 1 × 10^6^
ERM-FD102	1 × 10^3^, 1.5 × 10^3^ and 2 × 10^3^
Video acquisition	
Camera gain	250–400
Camera shutter	12 ms to 25 ms
Frame rate	25 frames/s
Capture time	60 s
Video analysis	
Detection threshold	4–10
Blur	Automatic adjustment
Minimum expected particle size	Automatic adjustment
Minimum track length	Automatic adjustment

## Conclusion

The PTA method has been fully validated for the determination of modal, median and arithmetic mean values of number-weighted PSDs. Various fit-for-purpose silica and polystyrene reference materials consisting of near-spherical and spherical particles were selected, and measurements were carried out according to a nested scheme. This approach allowed, via one-way ANOVA, to analyse the differences between and within measurement days, thereby effectively separating the variances from method repeatability and intermediate precision. In addition to precision, the trueness of the PTA method was assessed by analysing a monodisperse colloidal silica CRM under intermediate precision conditions.

The validation study included uncertainty estimation using a simple model where type A relative standard uncertainties for precision and trueness were combined. At the beginning of the study, the target relative expanded uncertainty was set to 10%. For measurements performed in the range of 50 nm to 100 nm (not too close to the lower LOQ, i.e. 41 nm), this criterion is met since uncertainties vary from 10% to 11%. Despite its inherent resolving power stemming from the particle-by-particle measurement principle, the PTA method lacks accuracy when determining the arithmetic mean values of particles whose sizes are close to the lower LOQ and PSDs that are skewed due to the presence of agglomerates. The modal and median measurands are found to be equally robust. Measurement uncertainties estimated from the PTA validation data agree well with uncertainties estimated previously by De Temmerman et al. (2014b). However, the PTA measurement uncertainties are a factor two larger than uncertainties for TEM and DLS measurement results (De Temmerman et al. 2014b; Braun et al. 2011).

The results and measurement uncertainties, presented in this contribution, may be used by other laboratories to help them in estimating the uncertainties of their own PTA results. To do so, the laboratory must ensure that their measurements are performed strictly within the scope of the validated method and that the validity of the modified Stokes-Einstein equation is not violated

## Electronic supplementary material

ESM 1 (DOCX 798 kb)
