# Supplementary material for: Validation of a particle tracking analysis method for the size determination of nano- and microparticles
Source: J Nanopart Res. 2017 Aug 4;19(8):271. doi: 10.1007/s11051-017-3966-8 (PMC5543194; doi:10.1007/s11051-017-3966-8)
Supplement: Supplementary file 1 — (DOCX 798 kb) [file 11051_2017_3966_MOESM1_ESM.docx]

Electronic Supplementary Information

**Validation of a particle tracking analysis method for the size determination of nano- and microparticles**

Vikram Kestens ^1*^, Vassili Bozatzidis ^1^, Pieter-Jan De Temmerman ^2^, Yannic Ramaye ^1^, Gert Roebben ^1^

^1^ European Commission, Joint Research Centre (JRC), Directorate Health, Consumers and Reference Materials, Retieseweg 111, B-2440 Geel, Belgium

^2^ Veterinary and Agrochemical Research Centre (CODA-CERVA), Service Trace Elements and Nanomaterials, Groeselenberg 99, B-1180 Brussels, Belgium

* Correspondence: Vikram Kestens, e-mail: [vikram.kestens@ec.europa.eu](mailto:vikram.kestens@ec.europa.eu), tel.: +32 14 571 614, fax: +32 14 517 548

**Method optimisation before validation**

The method validation process was preceded by an optimisation stage, which involved investigating the extent to which varying levels of selected method parameters can affect the ability to determine the characteristic values of the number-weighted size distributions of monomodal and bimodal mixtures of nano- and microparticles. The critical parameters investigated were: particle concentration, measurement temperature, and the camera parameters gain and shutter speed. The camera gain is a numerical setting that allows adjusting the sensitivity (signal-to-noise ratio), e.g., to detect smaller particles. The camera shutter determines the length of time during which the electronic shutter is open and thus how much light is captured from the particles. The video capture time was kept constant at 60 s. By increasing the capture time, more particles will be tracked. However, its downside is that the same particle may leave and re-enter the focal plane more than once thereby affecting the statistical reliability.

This optimisation stage, which mainly aimed to investigate and define the limitations of the PTA method must not be confused with the robustness study which is part of the method validation stage, where only the effect of defined limited changes is quantified.

*Particle concentration*

The influence of the particle concentration was investigated by analysing dilution series of PSL-100. The dilution series covered the concentration range which the instrument supplier recommends, i.e. between 10^7^ and 10^9^ particles/mL. These measurements were performed at 25 °C. As the samples were diluted in water, an associated viscosity of 0.889 mPa·s was used. The frame rate, camera gain and shutter speed, and detection threshold were set to 25 frames/s, 250, 1 ms to 12 ms, and 25 pixels respectively. The acquired video files were analysed with NTA 2.3 and NTA 3.0 software.

Fig. S1 illustrates the modal and arithmetic mean hydrodynamic diameters of PSL-100 as a function of the particle concentration. The second data layer of the graphs gives the number of valid tracks that were used for constructing the PSDs. The upper graph presents the results that were computed with the NTA 2.3 software. Within the recommended concentration range (the area between the vertical dashed lines) the mean and modal particle size results agree with the TEM reference value within ± 3 %. Beyond this concentration range, acceptable results were also obtained for particle concentrations down to 1.9 · 10^6^ particles/mL and up to 3.9 · 10^9^ particles/mL.

The lower graph of Fig. S1 presents data that were computed with the NTA 3.0 software. Overall, the trends between the calculated particle size results and the theoretical particle concentration are similar to the results from NTA 2.3. A good agreement between the modal and mean particle size values and the TEM reference value was found for the concentration range 1.9 · 10^6^ particles/mL to 1.9 · 10^9^ particles/mL. The modal values were sometimes just below the lower limit of 97 nm. However, it must be noted that the indicated measurement uncertainties are only the standard deviations calculated from the repeated results. If these uncertainties include contributions from intermediate precision and trueness, then a better overlap with the reference size range is expected.


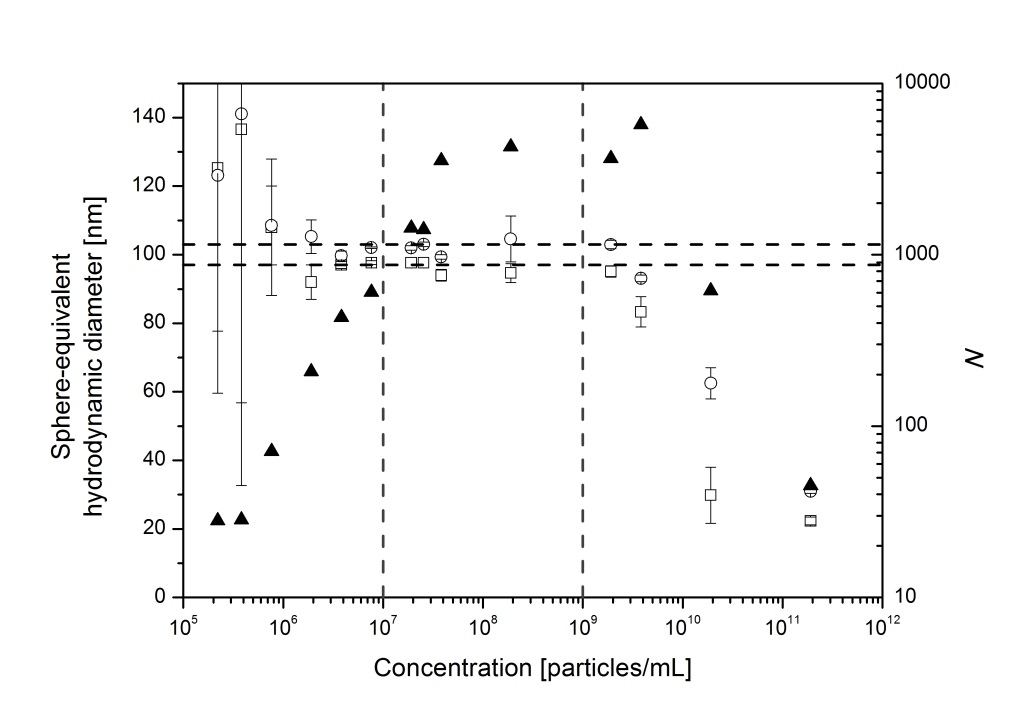


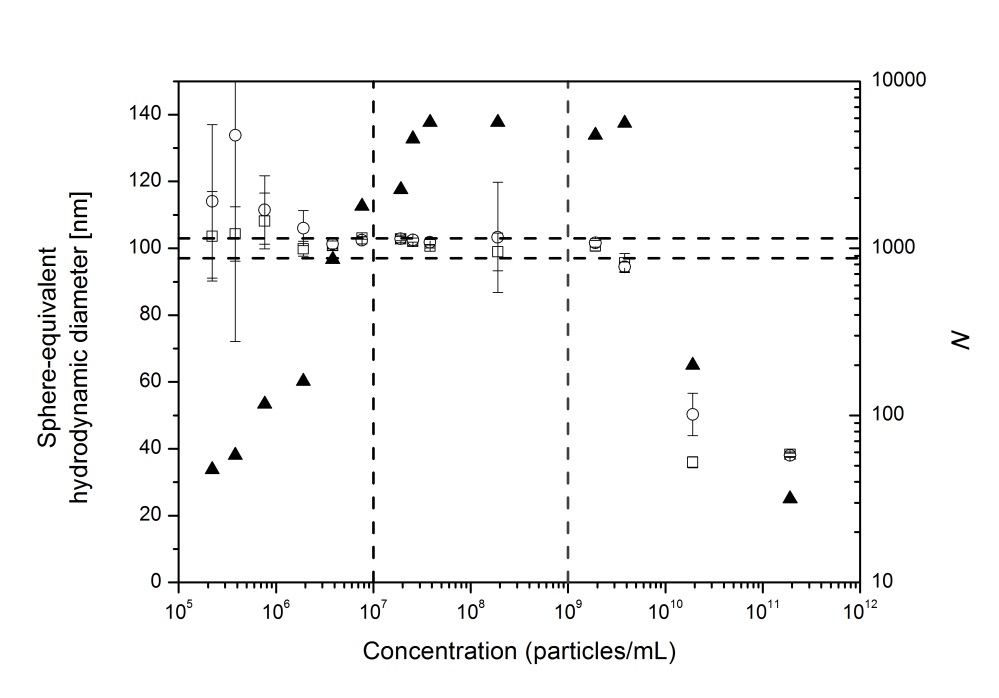


**Fig. S1** Influence of the theoretical particle concentration on the mean number (*N*) of valid tracks (triangles) and on the modal (squares) and arithmetic mean (circles) size values of number-weighted distributions of PSL-100. Particle size distributions were obtained using NTA 2.3 (upper graph) and NTA 3.0 (lower graph). Error bars correspond to the standard deviations calculated from replicate results of *n* = 3 (1.9 · 10^6^ particles/mL to 3.8 · 10^7^ particles/mL, and 3.8 · 10^9^particles/mL to 1.9×10^11^ particles/mL), *n* = 6 (1.9 · 10^8^ particles/mL and 1.9 · 10^9^ particles/mL) or *n* = 9 (2.2 · 10^5^ particles/mL to 7.6 · 10^5^ particles/mL). The vertical dashed lines are the upper and lower particle concentration limits as recommended by the instrument manufacturer. The horizontal dashed lines enclose the size range (100 nm ± 3 nm) assigned by the material supplier.

At high particle concentrations (1.9 · 10^10^ particles/mL and higher), PTA measurements were affected by multiple scattering phenomena and excessive overall drift velocities (1000 nm/s to 5000 nm/s). A high drift velocity causes the particles to move much faster than if they were under normal Brownian motion conditions. Because of the multiple scattering, individual particles could not be easily determined and this resulted in a reduced number of tracked particles. At low particle concentrations (7.6 · 10^5^ particles/mL and lower), the PSDs were not always monomodal but were often found to be polydisperse and sometimes even multimodal. The irregular shapes of the PSDs, which to a larger extent influence the mean particle size values than the modal values, were caused by the presence of few larger particles (e.g., small agglomerates of primary particles). These larger particles were sometimes also seen at higher concentration. However, at high concentration, the shape of the PSD is more robust because it mainly represents the abundantly present primary particles.

In summary, the results obtained show that for PSL-100 at a particle concentration range from 1.9 · 10^6^ particles/mL to 1.9 · 10^9^ particles/mL, both NTA 2.3 and NTA 3.0 software calculate mean and modal particle size results which are accurate within ± 1.5 %.

*Temperature*

Temperature and viscosity are two critical input parameters of the Stokes-Einstein equation. Changes in viscosity as a result of temperature fluctuations during a measurement can have a significant influence on the result. For that reason, PTA measurements should be performed under stable temperature conditions. The NS500 instrument is equipped with a Peltier element that allows setting the temperature within a range of 5 °C to 55 °C and controlling it with a precision better than 0.5 °C. The influence of the set temperature (20 °C to 30 °C) on the mean and modal particle size was investigated for PSL-100 at a concentration of 1.9 · 10^7^ particles/mL. The frame rate, camera gain, shutter speed and detection threshold were set to 25 frames/s, 250 to 320, 12 ms, and 25 pixels respectively. The acquired video files were analysed with NTA 2.3 and NTA 3.0 software. Viscosity settings for water were applied and automatically corrected for the measured temperature.

Fig. S2 shows that the calculated particle size and the measurement temperature are linearly correlated. Significant differences between the modal and the mean particle sizes were seen for results analysed with NTA 2.3 while the results of the two measurands better agree when analysed with NTA 3.0. It was noticed that the temperature inside the measurement chamber could increase to 35 °C due to the heat dissipated by the electronic components. A measurement temperature of 35 °C was not considered during the experiments because the results (for NTA 3.0) obtained at 28 °C, and higher were already above the reference particle size range of 97 nm to 103 nm. On the other hand, the modal results obtained at temperatures below 24 °C and which were computed with NTA 2.3 were below the lower limit of the reference size range.

In considering the two particle size measurands and the two instrument software programmes, it can be concluded that the instrument's operating temperature must be restricted to the range of 24 °C to 26 °C to provide reproducible and reliable results. This is an unfortunate conclusion, but it is within the instrument's capacity to provide such a level of temperature control.


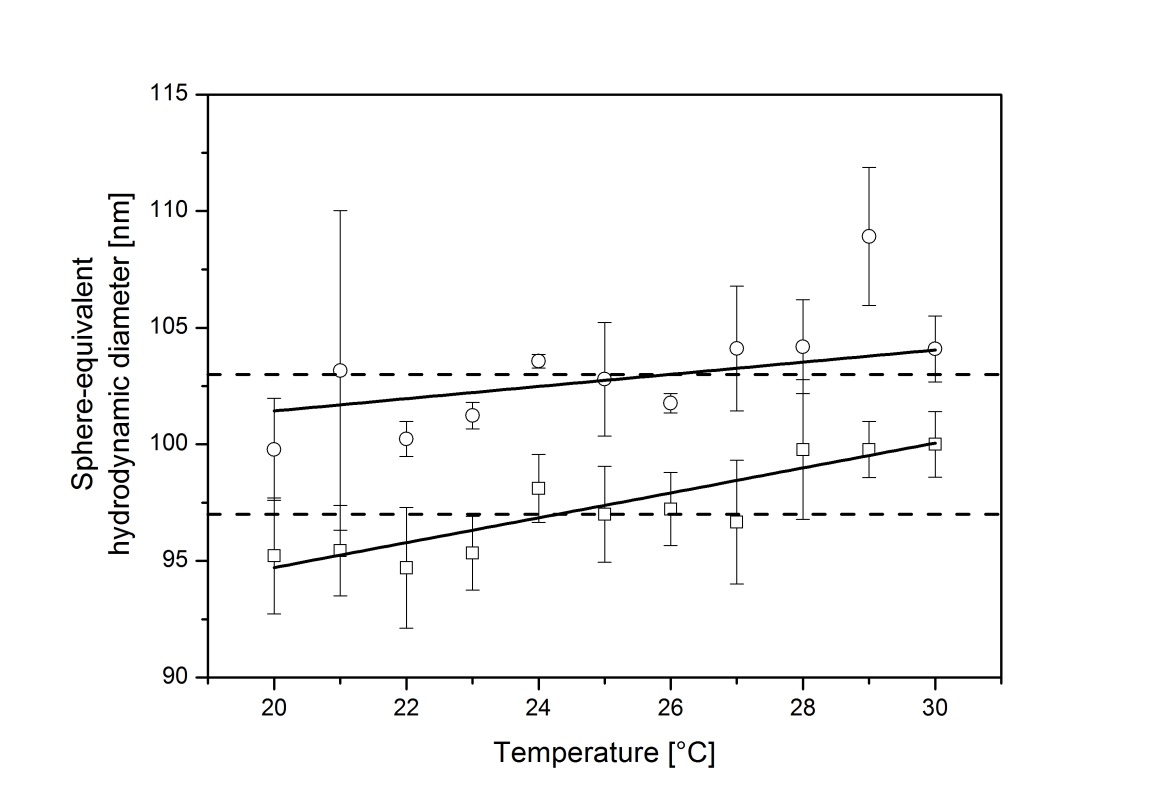


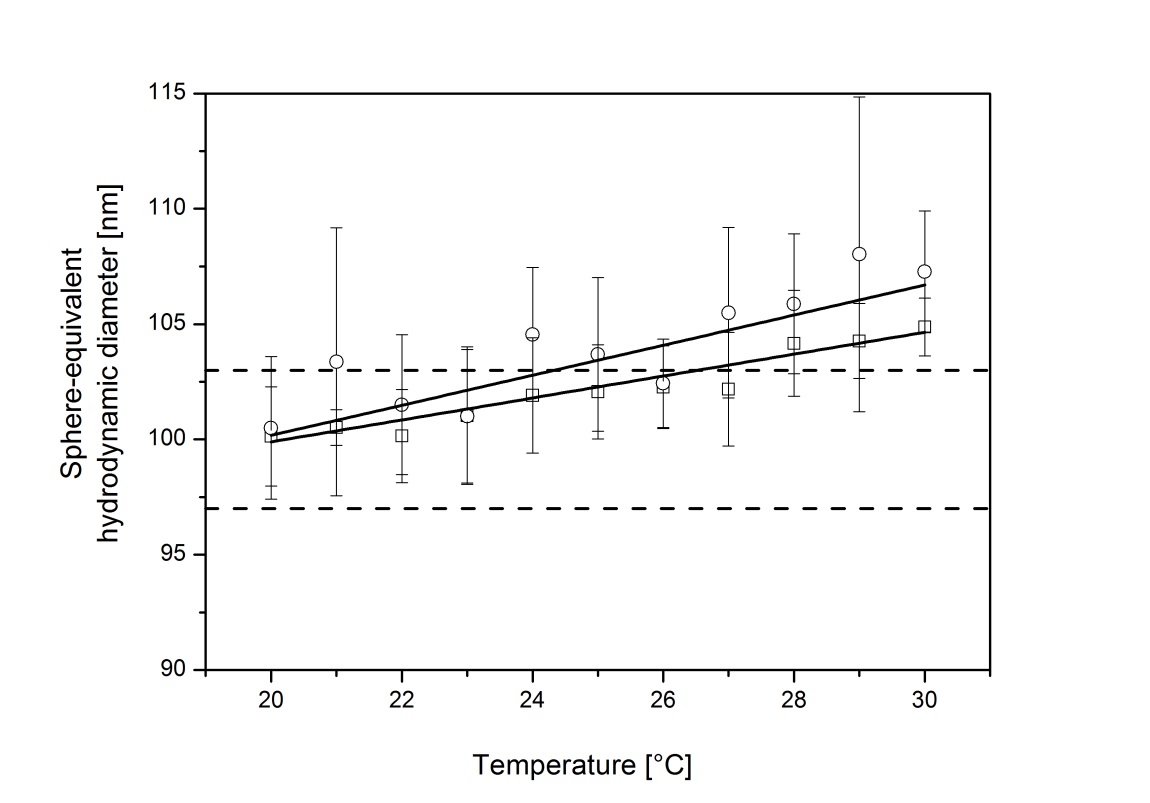


**Fig. S2** Influence of the set temperature on the modal (squares) and arithmetic mean (circles) size values of number-weighted distributions of PSL-100. Measurements were obtained in a randomised order, and particle size distributions were computed using NTA 2.3 (upper graph) and NTA 3.0 (lower graph). Error bars correspond to the standard deviations calculated from replicate results (*n* = 9). The horizontal dashed lines enclose the size range (100 nm ± 3 nm) assigned by the material supplier.

*Camera gain and shutter speed*

The next step in the method optimisation process was to define the optimal video acquisition settings for the gain and shutter speed of the camera. The influence of both parameters was investigated by analysing a test sample of PSL-100 at 1.9 · 10^7^ particles/mL. All measurements were performed under repeatability conditions at 25 °C. The frame rate and detection threshold were set to 25 frames/s and 25 pixels respectively. When varying the levels of the camera gain, the shutter speed was fixed to a value of 12. On the other hand, when varying the levels of the shutter speed, the camera gain was fixed to its default value of 250. The acquired video files were analysed with NTA 2.3 and NTA 3.0 software.

As can be seen from Fig. S3, the overall trend is that the calculated particle size value decreases with increasing camera gain. This trend is more pronounced for results computed with software NTA 2.3 (upper graph) than for results from NTA 3.0 (lower graph). The highest degree of agreement is seen for results obtained at camera gain values ranging from about 100 to 300. A camera gain value of 250 is used by default by the instrument software. Despite the correlation trend, for each measurand the results agree within their stated measurement uncertainties. As a result, it can be concluded that for the tested material the camera gain has only a limited effect on the particle size.

No correlation between particle size and camera shutter speed was observed, neither for data analysed using NTA 2.3 nor for data analysed by software NTA 3.0 (Fig. S4).


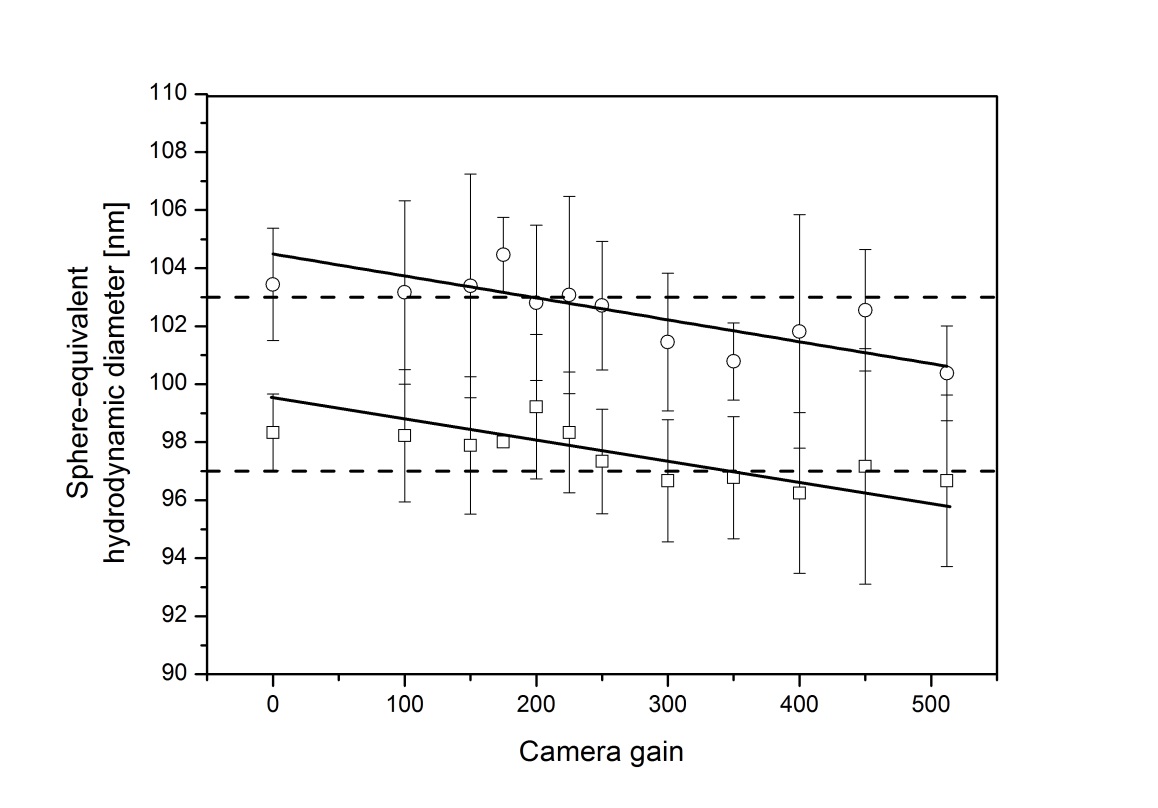


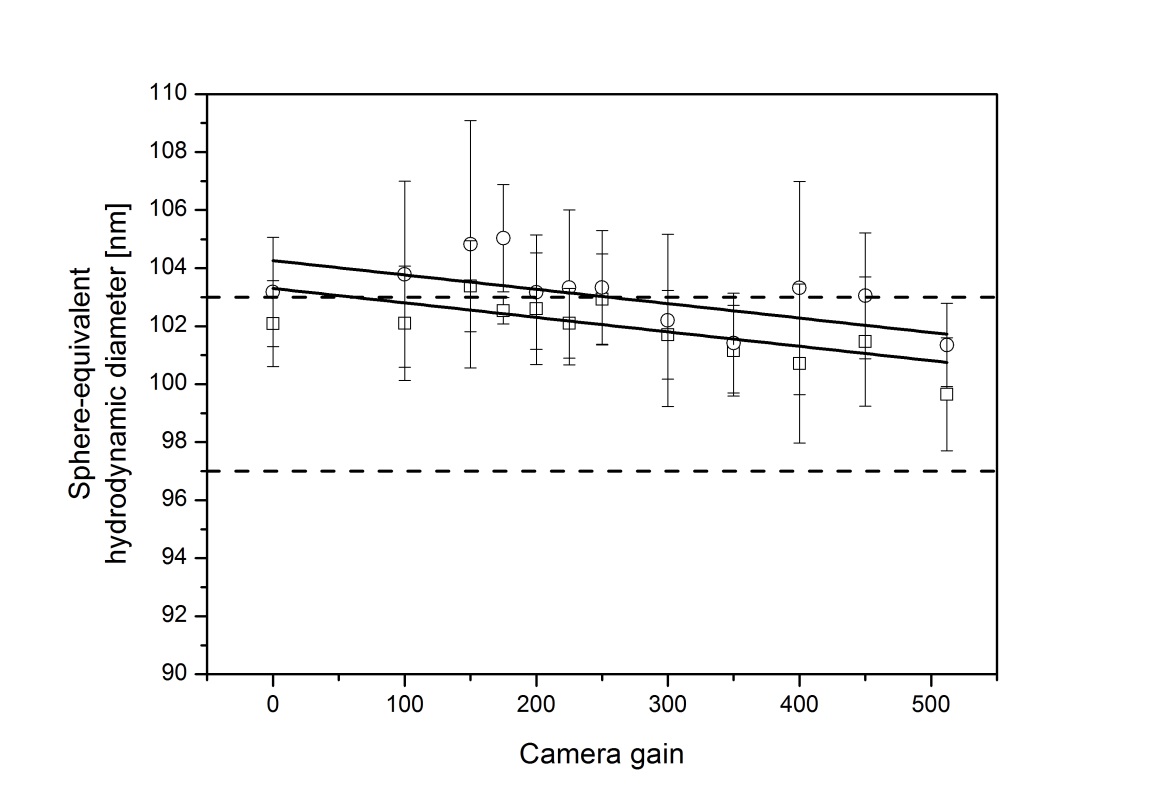


**Fig. S3** Influence of the camera gain on the modal (squares) and arithmetic mean (circles) size values of number-weighted distributions of PSL-100. Particle size distributions were obtained using NTA 2.3 (upper graph) and NTA 3.0 (lower graph). Error bars correspond to the standard deviations calculated from replicate results of *n* = 9. The horizontal dashed lines enclose the size range (100 nm ± 3 nm) assigned by the material supplier.


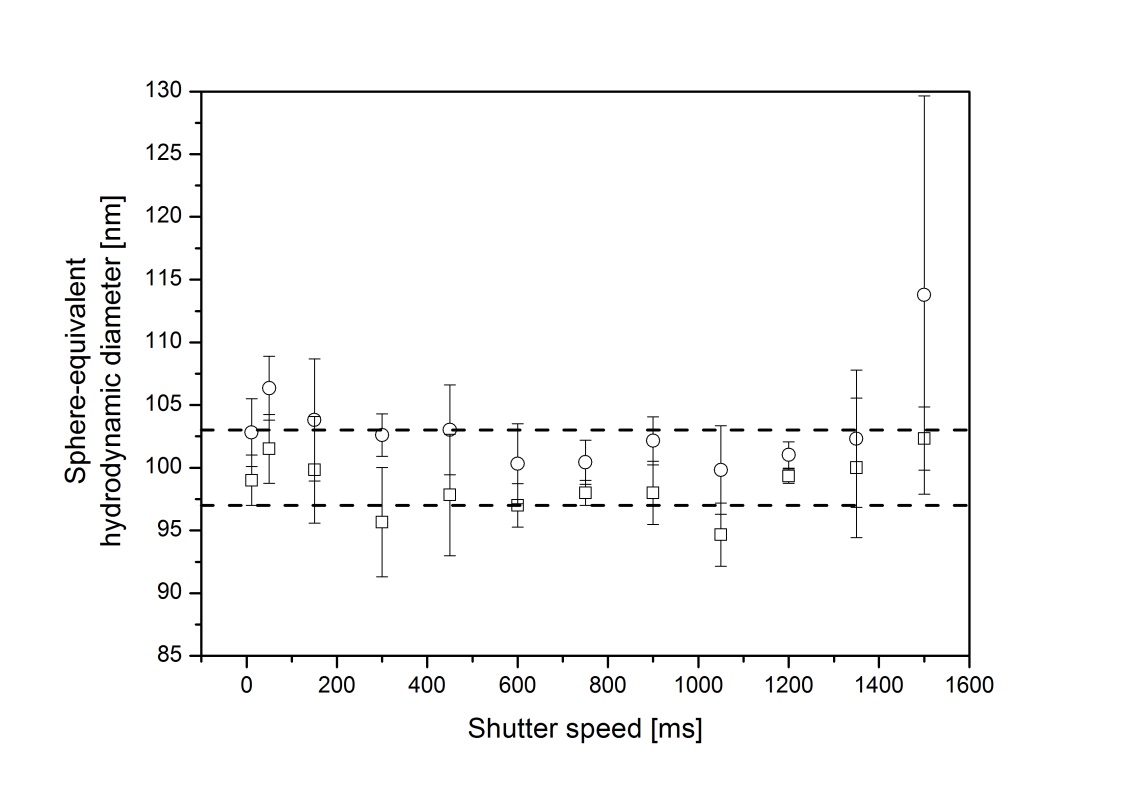


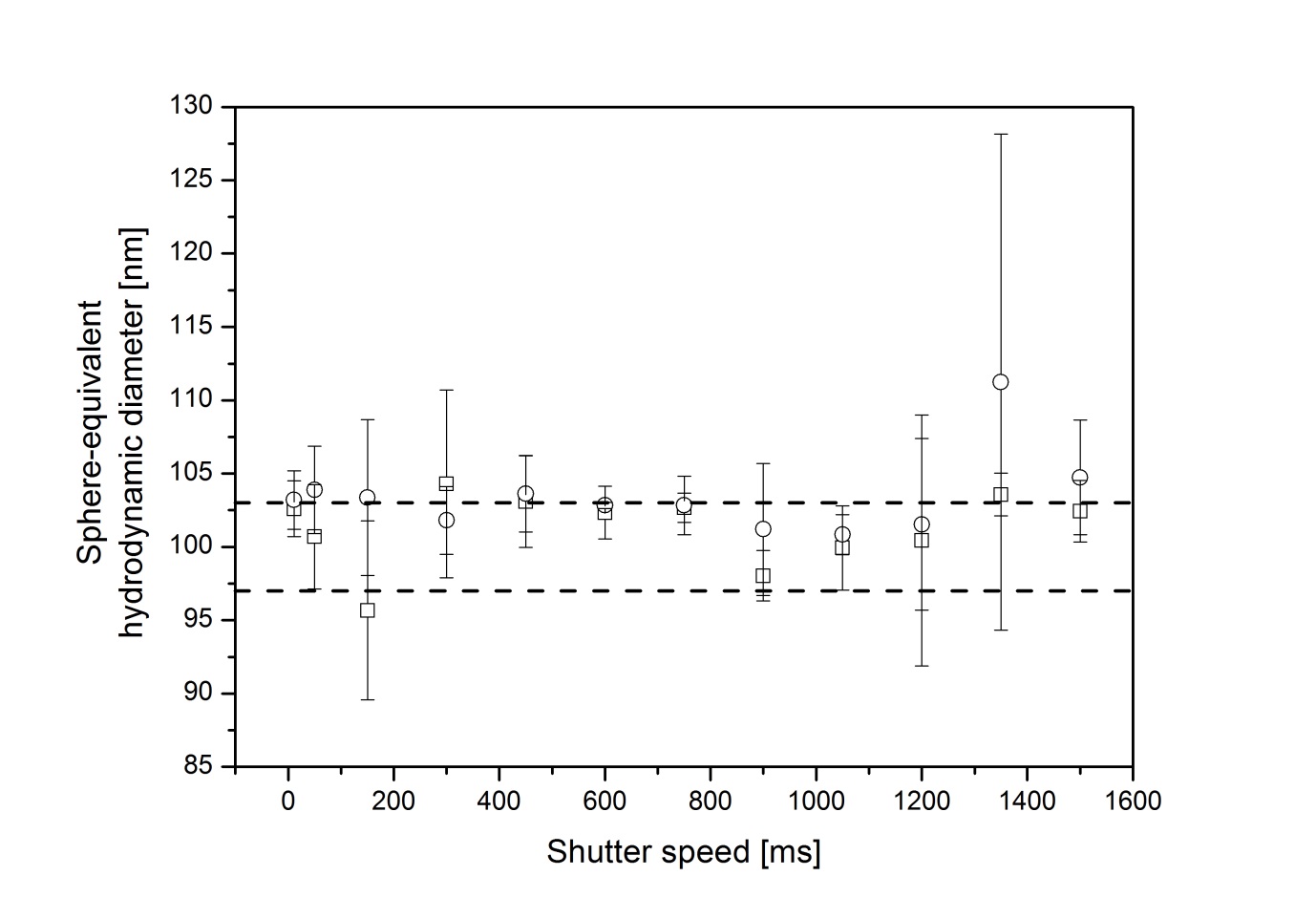


**Fig. S4** Influence of the camera shutter speed on the modal (squares) and arithmetic mean (circles) size values of number-weighted distributions of PSL-100. Particle size distributions were obtained using NTA 2.3 (upper graph) and NTA 3.0 (lower graph). Error bars correspond to the standard deviations calculated from replicate results (*n* = 3) for data at 600 ms to 1500 ms; *n* = 6 for data at 50 ms to 450 ms. The horizontal dashed lines enclose the size range (100 nm ± 3 nm) assigned by the material supplier.

**Estimation of method resolution**

The resolution (*R*_s_) is a measure of how well two neighbouring peaks are separated. In this section, the different approaches which originate from chromatography data analysis (Snyder 2010) are briefly discussed and applied to the PTA number-weighted PSDs of bimodal PSL mixtures.

For baseline-resolved peaks, the following equation (Eq. S1) is commonly used:

$R_{s}= \frac{X_{c2}- X_{c1}}{0.5 \cdot(W_{1}+ W_{2})}$ (Eq. S1)

where, *X*_c1_ and *X*_c2_ correspond to the local maxima of peak 1 and peak 2, and *W*_1_ and *W*_2_ are the full widths of the two peaks measured at their baseline.

Often, peaks are not fully resolved. In this case, the full widths (at the baseline) of the marginally separated peaks cannot be directly determined. An acceptable alternative is to use the full-width at half-height. The *R*_s_ value can then be calculated according to Eq. S2:

$R_{s}= \frac{X_{c2}- X_{c1}}{1.7 \cdot0.5 \cdot(W_{0.5,1}+ W_{0.5, 2})}$ (Eq. S2)

where, *W*_0.5,1_ and *W*_0.5,2_ are the peak widths measured at half-height, the value of 1.7 is a factor which adjusts for the difference in width at the half-height.

For Gaussian peaks, the *R*_s_ values calculated with Eq. S1 and Eq. S2 will be the same. If the valley of two adjacent peaks touches the baseline, then the *R*_s_ value will be ≈ 1.5. When the resolution further drops, the valley between the two peaks rises and at a certain point the full width at half-height of the overlapping peaks can only be estimated by curve fitting.

For the presented PTA validation study, the characteristic value of the number-weighted PSDs which is of primary interest, is the modal value, in the case of a monomodal distribution, or the local maxima, in the case of a multimodal distribution. The reported *R*_s_ values were calculated according to Eq. S2. The necessary peak characteristic values were estimated by fitting the experimental data of the two individual peaks with simulated monomodal data that followed a Gaussian function (Fig. S5).


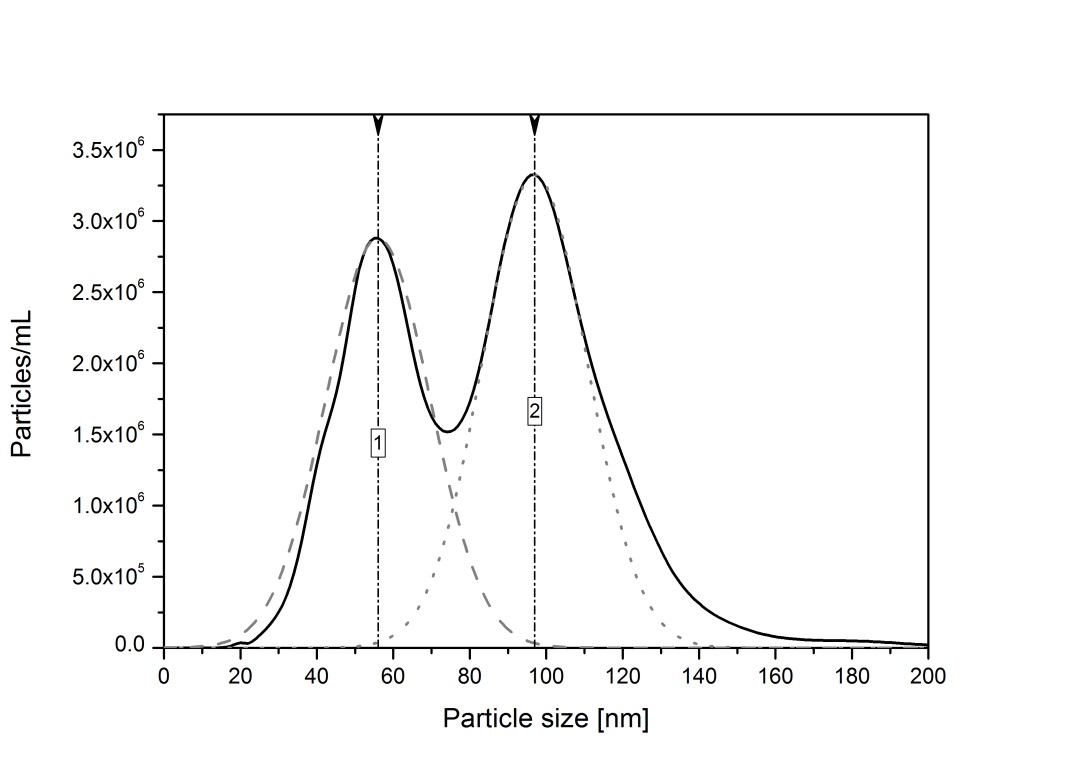


**Fig. S5** Bimodal number-weighted PSD of PSL-50-100_2:1 obtained using NTA 2.3; partly resolved peaks are fitted individually with simulated monomodal distributions (dashed and dotted curves) following a Gaussian function.

**References**

Snyder LR, Kirkland JJ and Dolan WJ (2010) An introduction to modern liquid chromatography. 3^rd^ edition John Wiley & Sons, Inc. Hoboken, New Jersey
